# A Case of Large Subchorionic Hematoma

**DOI:** 10.7759/cureus.99263

**Published:** 2025-12-15

**Authors:** Igor Lakhno, Andriy Tkachov, Serhiy Korovai

**Affiliations:** 1 Department of Obstetrics and Gynecology, Kharkiv National Medical University, Kharkiv, UKR; 2 Department of Ultrasound Diagnosing, Kharkiv Municipal Perinatal Center, Kharkiv, UKR; 3 Department of Gynecology, Kharkiv Municipal Perinatal Center, Kharkiv, UKR

**Keywords:** breus' mole, obstetric hemorrhage, preterm birth, preterm placental abruption, subchorionic hematoma

## Abstract

The cases of persistent subchorionic hematoma are not rare. The management includes the conventional approaches to the treatment of threatened miscarriage. This case demonstrates a large subchorionic hematoma as a cause of preterm placental abruption and preterm birth via cesarean section.

We present the case of a 23-year-old G1P0 patient with a massive subchorionic hematoma during the first, second, and third trimesters. The woman was admitted to the gynecological clinic several times because of threatened miscarriages at 9, 11, 13, and 20 weeks of pregnancy. Since then, multiple subchorionic or intermembranous hematomas were visualized on ultrasound. They were resistant to progesterone and tranexamic acid. However, hemodynamic Doppler ultrasound did not reveal any abnormalities of the utero-placental, umbilical, or fetal hemocirculation later on. Fetal growth parameters were appropriate. The patient was admitted with bleeding due to preterm placental abruption at 35 weeks of gestation. A live preterm male baby was born via urgent cesarean, weighing 2300 g, measuring 46 cm in length, with an Apgar score of 6-7. Placental histological examination revealed a massive placental thrombohematoma (Breus’ mole). The mother had a shortened pQ interval on the ECG tracing and an incomplete uterine septum. A true umbilical knot was found. The newborn passed through the neonatal resuscitation unit. After 10 days, both the mother and her baby were released from the hospital.

A persistent subchorionic hematoma and prolonged mild bloody discharge raise suspicion for Breus’ mole. The initial venous source hemorrhage triggered the rupture of arterial utero-placental vessels. Maternal cardiac events, uterine and umbilical anomalies were involved in this case. Preterm placental abruption occurred because of placental thrombohematoma.

## Introduction

Subchorionic hematoma and vaginal bleeding are signs of threatened abortion [1]. Treatment options include the use of progesterone and tranexamic acid. Pregnancy may be interrupted spontaneously or prolonged if the hematoma reduces. However, hematoma persistence is not rare. Inherited or acquired thrombophilia is a known cause of subchorionic hematoma [2]. The massive placental thrombohematoma, or Breus’ mole, is a deposition of a large clot at the site of umbilical cord insertion into the placenta. Placental thrombohematoma was first described as Breus's mole in 1892. The frequency of this pathology is only 0.03-0.08%, and its etiology and pathogenesis are still unknown [3]. A massive placental hematoma can deteriorate umbilical hemodynamics. This abnormality is a serious complication, often related to fetal growth restriction or antenatal fetal death. Sometimes, a subchorionic hematoma found in the first trimester can persist into the second and third trimesters, leading to preterm placental abruption [4]. Managing patients with persistent hematoma or partial non-progressive placental abruption is very important. The conventional red line in obstetrical practice is the possibility of conservative management in preterm placental abruption. This pathology is associated with fetal distress, severe maternal hemorrhage, disseminated intravascular coagulation, and Couvelaire uterus. However, successful prolongation of pregnancy is possible when the area of abruption is small, fetal well-being is normal, and severe symptoms - such as an increased uterine tone, abnormal maternal hemodynamics, or heavy bloody discharge - are absent [5]. This case demonstrates persistent subchorionic hematoma as a cause of preterm placental abruption and preterm birth via cesarean section.

## Case presentation

**Figure 1:**
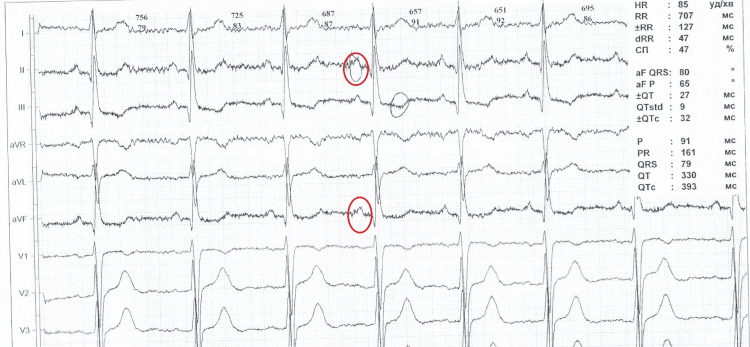
Maternal ECG tracing The shortened pQ interval is visible. Abnormal p-peaks painted in red circles.

**Figure 2:**
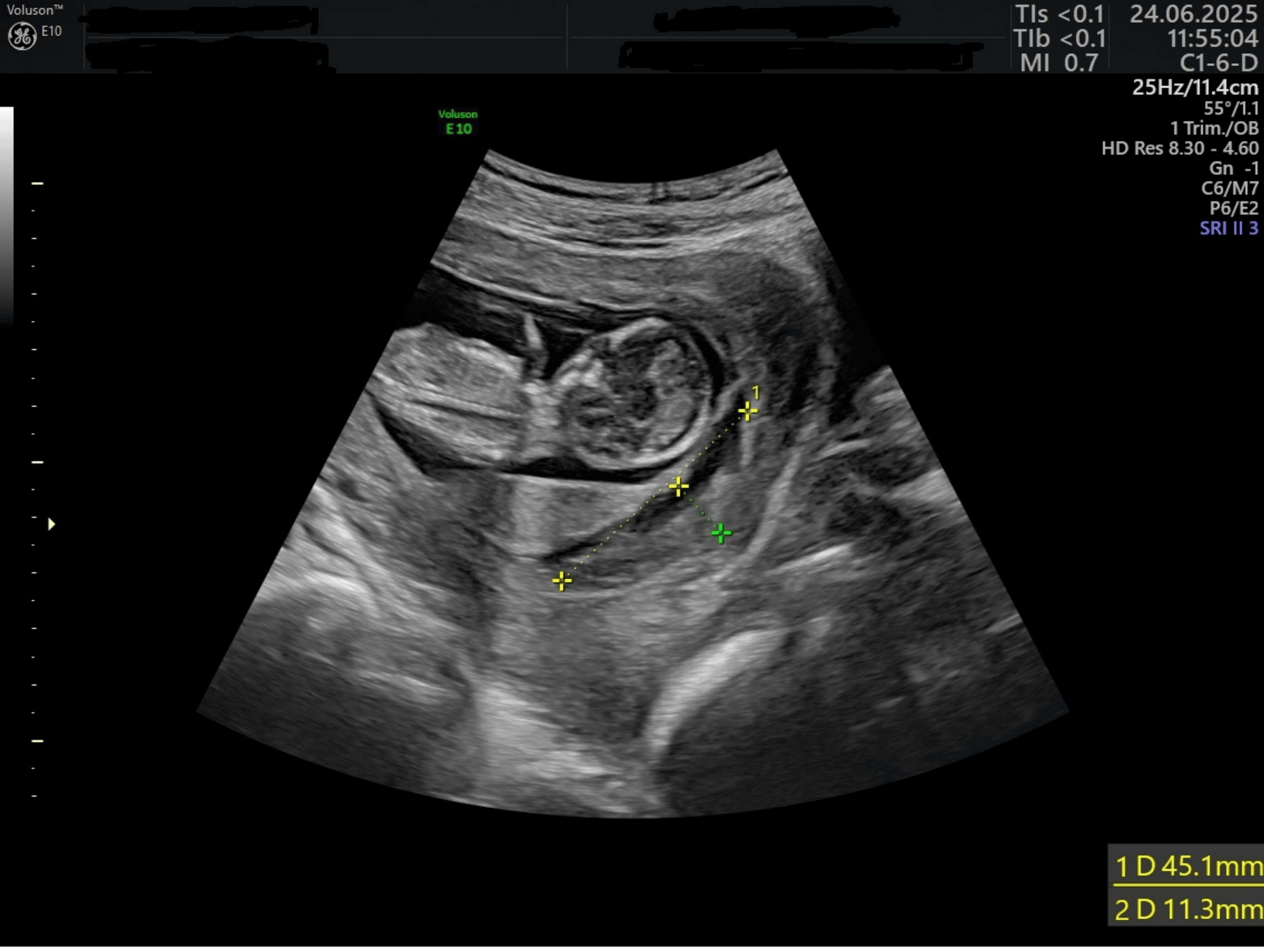
A subchorial hematoma A large subchorial hematoma is visible.

A 23-year-old patient had her initial antenatal visit to an outpatient clinic at six weeks of gestation. She was G1P0, and her general medical history was unremarkable. The patient has a uterine anomaly - an incomplete septum. Management was conducted in accordance with current standards. The ECG showed a decreased pQ interval (Figure 1). The woman was hospitalized in the gynecological department of the Kharkiv Municipal Perinatal Center because of threatened miscarriages at 9, 11, 13, and 20 weeks of pregnancy. The results of the infectious screening were negative. An increased level of IgG to cytomegalovirus was detected. Biochemical screening revealed no abnormalities. Ultrasound screenings did not reveal any evidence of genetic abnormalities. Nevertheless, Doppler ultrasound revealed placental dysfunction. At the end of the first and beginning of the second trimester, multiple subchorionic or intermembranous hematomas were visualized on ultrasound, indicating a threat of miscarriage (Figure 2). A placental thickening was also detected. Examination for antiphospholipid syndrome excluded this pathology. She received multivitamin complexes with trace elements, low doses of acetylsalicylic acid, progesterone preparations, sorbitol for small-volume infusion therapy, and L-arginine solution. The fetal growth parameters detected via ultrasound and hemodynamic Doppler indices were normal at 26 and 32 weeks.

**Figure 3:**
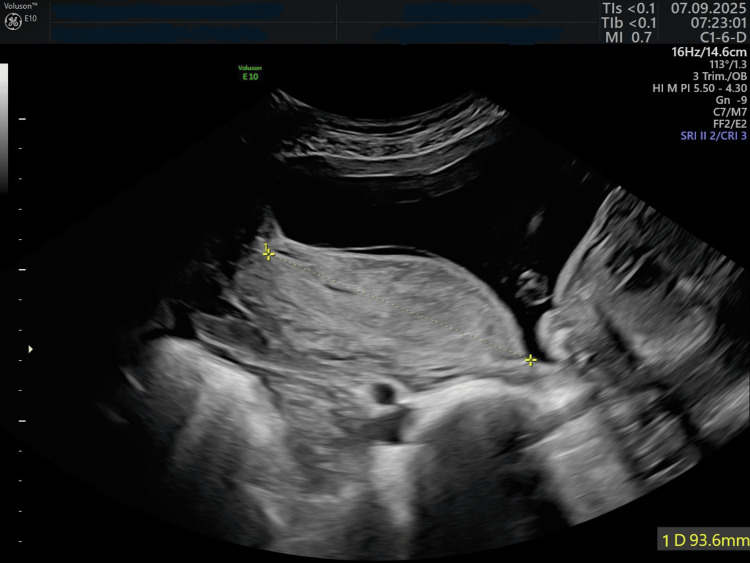
Preterm placental abruption A marginal preterm placental abruption is visible.

**Figure 4:**
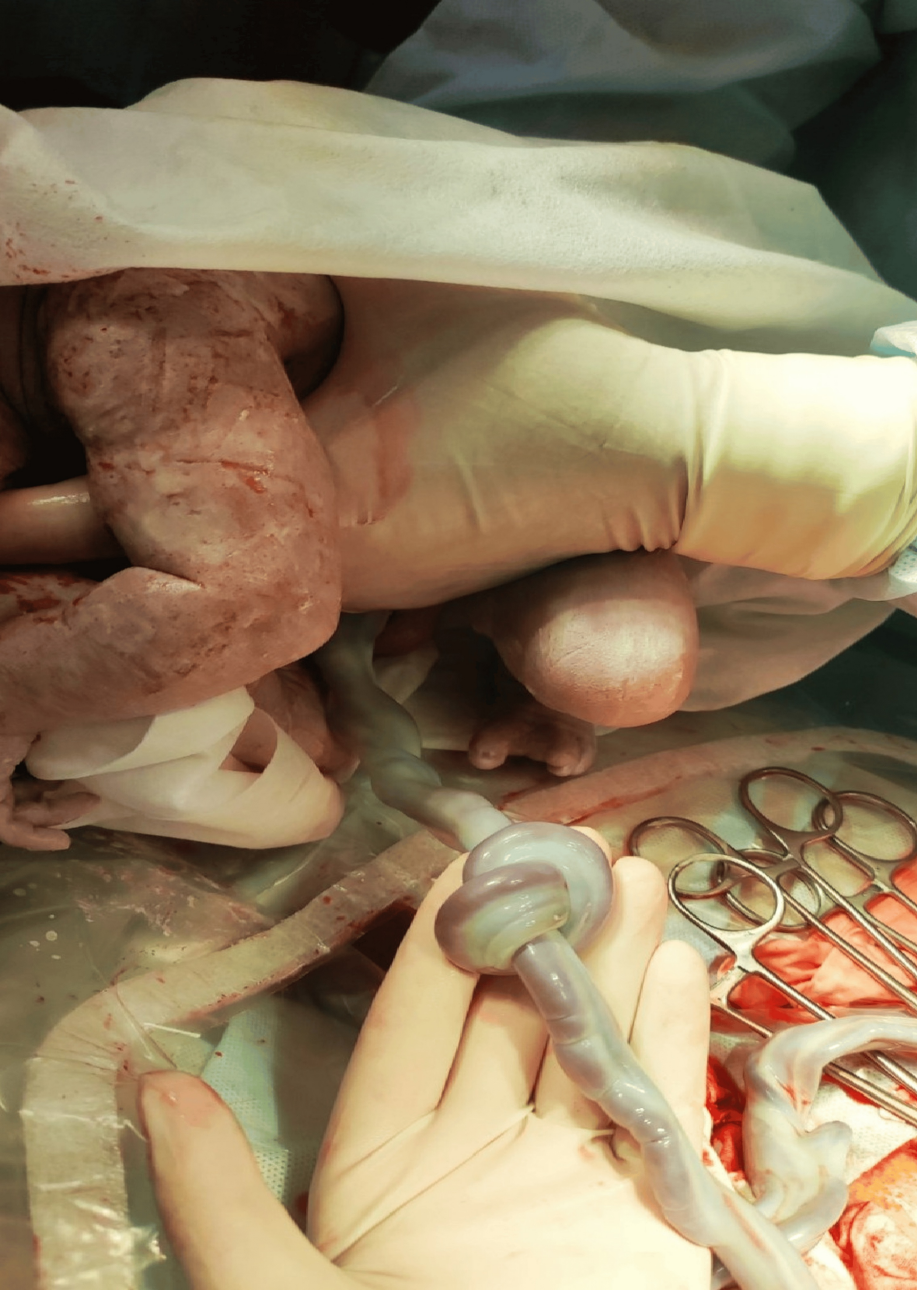
A true umbilical knot A true umbilical knot is visible.

The patient was admitted with bleeding due to preterm placental abruption at 35 weeks of gestation. She was hemodynamically stable, and the fetal heart rate was normal. Ultrasound imaging (Figure 3) confirmed the diagnosis of marginal placental abruption. An urgent cesarean section was performed. A live preterm male baby was born weighing 2300 g, measuring 46 cm in length, with an Apgar score of 6-7. A true umbilical knot was found (Figure 4). Placental histological examination revealed a massive placental thrombohematoma (Breus’ mole). The dynamic of the laboratory tests is presented in Table 1. The newborn was admitted to the neonatal resuscitation unit. After 10 days, both the mother and her baby were released from the hospital.

**Table 1:** A laboratory tests in the mother The dynamics of general laboratory tests during second, third trimesters and at discharge is presented.

Laboratory test	20 weeks	35 weeks	At discharge	Normal range
WBC	10.2×10^9^/L	11.5×10^9^/L	9.8×10^9^/L	<14×10^9^/L
Hb	115 g/l	112 g/l	110 g/l	>110 g/l
CRP	0.8 mg/dl	0.9 mg/dl	0.6 mg/dl	<1 mg/dl
Total protein	72 g/l	69 g/l	70 g/l	60-83 g/l
Urea	5.1 g/l	6.2 g/l	4.0 g/l	2.1–8.5 mmol/L
Creatinine	0.6 mg/dl	0.8 mg/dl	0.7 mg/dl	0.6 to 1.1 mg/dL
Urinalysis	Unremarkable	Unremarkable	Unremarkable	Unremarkable

## Discussion

This case was atypical. Higher rates of maternal heart conditions, uterine anomalies, thrombophilia, and abnormalities in the placenta and umbilical cord were observed among patients with a Breus’ mole. An ECG revealed a shortened pQ interval in the mother; an incomplete uterine septum was found, and a true umbilical knot was identified. Information regarding inherited or acquired thrombophilia was not collected. The subchorionic hematoma found in the first trimester caused a marginal preterm placental abruption later, at 35 weeks of gestation. The bleeding was mild, as it was of a venous nature. Subchorionic thrombohematoma is a rare condition in which a large maternal blood clot separates the chorionic plate from the villous chorion. The hematoma is typically located at the site of umbilical cord insertion. It can cause cord compression, umbilical vein obstruction, and decreased fetal perfusion [6]. However, it did not cause fetal growth restriction. Placental thickening increases the suspicion of a Breus' mole. Ultrasound imaging does not readily differentiate between hematoma and placental tissue [7]. Prolonged use of progesterone and tranexamic acid did not reduce the hematoma. Previous considerations revealed an increased risk of gestational complications and poor perinatal outcomes in the case of a Breus’ mole [3].

It was not possible to predict preterm placental abruption ahead of time. Failed placentation has obvious hemodynamic Doppler features. The uteroplacental, umbilical, and fetal hemodynamics were normal. The mechanisms of decidual bleeding in preterm placental abruption are related to the rupture of utero-placental vessels [8]. This bleeding is arterial. Therefore, the initially venous bleeding due to a massive thrombohematoma became of a mutual nature by adding arterial sources during the placental abruption. In this case, it is possible that preterm placental abruption had an inflammatory origin. The persistent hematoma and prolonged bloody discharge triggered the ascending way of infection and coexisting inflammation. The use of antibiotics was logical at the time. Preterm placental abruption is known as a complication of pre-eclampsia. Nonetheless, our patient did not develop pre-eclampsia. The sFLT/PlGF measurement was not performed [9]. The marginal preterm placental abruption could have a similar ultrasonic appearance to chorioangioma. The placental mass becomes evident as early as the first trimester. Chorioangioma is associated with preterm birth, pre-eclampsia, polyhydramnios, and fetal anemia [10].

## Conclusions

A persistent subchorionic hematoma, despite conventional treatment, and placental thickening may raise suspicion for Breus' mole. Further management should include serial hemodynamic Doppler to prevent poor perinatal outcomes. The abnormal findings on maternal ECG, incomplete uterine septum, and a true umbilical knot were presented in the case. Preterm placental abruption occurred because of placental thrombohematoma. The cesarean section and preterm birth were the outcomes of Breus' mole.
